# Asymmetric visual representation of sex from facial appearance

**DOI:** 10.3758/s13423-022-02199-6

**Published:** 2022-10-21

**Authors:** Marco Gandolfo, Paul E. Downing

**Affiliations:** 1 Donders Institute for Brain, Cognition, and Behaviour, Radboud University, Nijmegen, The Netherlands; 2 School of Human and Behavioural Sciences, Bangor University, Bangor, UK

**Keywords:** Social vision, Face perception, Sex perception, Visual search, Search asymmetry

## Abstract

We efficiently infer others’ traits from their faces, and these inferences powerfully shape our social behaviour. Here, we investigated how sex is represented in facial appearance. Based on previous findings from sex-judgment tasks, we hypothesized that the perceptual encoding of sex is not balanced but rather polarized: for the processes that generate a sex percept, the default output is “male,” and the representation of female faces extends that of the male, engaging activity over unique detectors that are not activated by male faces. We tested this hypothesis with the logic of Treisman’s studies of visual search asymmetries, predicting that observers should more readily detect the presence of female faces amongst male distractors than vice versa. Across three experiments (*N* = 32 each), each using different face stimuli, we confirmed this prediction in response time and sensitivity measures. We apply GIST analyses to the face stimuli to exclude that the search asymmetry is explained by differences in image homogeneity. These findings demonstrate a property of the coding that links facial appearance with a significant social trait: the female face is coded as an extension of a male default. We offer a mechanistic description of perceptual detectors to account for our findings and posit that the origins of this polarized coding scheme are an outcome of biased early developmental experience.

**Supplementary Information:**

The online version contains supplementary material available at 10.3758/s13423-022-02199-6.

## Introduction

Research in social vision seeks to understand the visual processes underpinning everyday social behaviour. Many studies have sought to understand how observers extract and use the socially relevant cues about others’ age, race, sex, health, emotion, direction of attention, and other states and traits that are visible in facial appearance (Adams et al., 2010; Todorov, 2017). Among these, the perception of sex^1^ has been of particular interest. Sex is considered one of the “big three” social dimensions on which observers tend to categorize others at first encounter (along with age and race; Carter, 1944; Ito & Urland, 2003). Evolutionary and social psychology suggest drivers that encourage categorization by sex, for example evaluating the fitness of potential mates (Little et al., 2001; Perrett et al., 1998), or activating associated information in semantic memory (Eagly & Mladinic, 1989; Skrypnek & Snyder, 1982; Stroessner, 1996). Here, we report new findings showing that the visual coding of sex from adult faces is not balanced symmetrically, but rather polarized, such that “female” is coded as an extension of a “male” default.

Adult human faces are sexually dimorphic with respect to shape, texture, and colouration. Accordingly, behavioural tests reveal observers’ capacity to judge sex on the basis of face properties including overall shape (Bruce et al., 1993; Nestor & Tarr, 2008), contrast (Russell, 2009; Russell et al., 2017), and pigmentation (Bruce & Langton, 1994; Nestor & Tarr, 2008), and from patterns of facial motion (Berry, 1991). Disrupting holistic processing or configural information interferes with sex judgments, suggesting a contribution of whole-face representations (Baudouin & Humphreys, 2006; De Gutis et al., 2012; Zhao & Hayward, 2010). Yet reliable sex judgments are also possible from individual face parts (Brown & Perrett, 1993; Schyns et al., 2002; Yamaguchi et al., 2013) or reveal a strong reliance on specific parts (Dupuis-Roy et al., 2009; Faghel-Soubeyrand et al., 2019; Macrae & Martin, 2007; Schyns et al., 2002). Finally, adaptation to a face of one sex shifts the subjective male/female boundary, so that subsequent ambiguous faces favour the other sex (e.g., Webster et al., 2004). Face adaptation persists over manipulations of the relative position, orientation, and size of adapter and test faces, excluding explanations based on low-level visual mechanisms (Afraz et al., 2010; Bestelmeyer et al., 2008; Webster & MacLeod, 2011). Together, this evidence shows that cues to sex are multiplexed in the appearance of the face, and high-level, face-specific representations offer several routes for an observer to make a sex judgment.

One key finding is that observers tend to default to a “male” judgment, especially when information about the face is ambiguous. The male bias is found in judgments of photographs (Watson et al., 2016), artificial faces (Armann & Bülthoff, 2012), face profile silhouettes (Davidenko, 2007), and illusory faces (Wardle et al., 2022), and for adults’ judgments of both child and infant faces (Boisferon et al., 2019; Tskhay & Rule, 2016). This pattern appears in several tasks, such as binary male/female choices, continuous judgments (how male/female?), or comparisons (which is more male?) about faces drawn from a morph series (e.g., Armann & Bülthoff, 2012; Graf & Wichmann, 2002; Watson et al., 2016; Wild et al., 2000).

An evolutionary account of the male bias emphasizes the survival implications of errors in person categorization: If unknown males are more likely to present a physical threat than females, then it may be less risky to err in favour of judging male (Haselton et al., 2015; Haselton & Buss, 2000). Instead from a mechanistic perspective, which is our focus here, one possibility is that the bias results from postperceptual decision-making: Visual representations of the face are balanced with respect to sex, and the male bias is introduced when a judgment is required. Here, we test the deeper possibility, that the male bias reveals asymmetries in the perceptual coding of the face: a polarized rather than a balanced representation (cf. Proctor & Cho, 2006; Watson et al., 2016). That is, for the mental processes that encode sex, the default output is “male,” and “female” is determined only in the presence of additional perceptual evidence. This implies that while male and female faces share many properties in common, the female face is positively coded by additional features or properties, relative to the male (cf. Wardle et al., 2022). Conversely, males, as the default percept, have fewer additional unique features that distinguish them from females. Importantly, we refer to features not in the everyday sense of face parts, but rather in terms of the components of mental representations.

To test this proposal, we adopted the approach of Treisman’s search asymmetry studies (Treisman & Gormican, 1988; Treisman & Souther, 1985). Visual search performance is sometimes asymmetrical, depending on whether a given item is a target or a distractor. For example, converging lines are found more efficiently amongst parallel pairs than vice versa, and search for an ellipse amongst circles is more efficient than the converse. Treisman suggested that the coding of some visual dimensions is organized around canonical values and extensions of those values. An ellipse, for example, is encoded as an extension of a canonical circle. While both kinds of stimuli activate detectors for the canonical property (e.g., circularity), deviations are further positively coded by additional activity over selective detectors that are not tuned to the default property. The asymmetry in search performance favouring deviating targets arises because it reflects a presence (an increment in activity) which is more readily detected than an absence (Neisser, 1963; Rajsic et al., 2020).

Similar logic has been adapted to understand the encoding of complex emergent stimulus properties (Enns & Rensink, 1990, 1991; Hulleman et al., 2000; Kristjánsson & Tse, 2001; Sun & Perona, 1996a, 1996b), including properties of the face (Becker et al., 2011; Becker & Rheem, 2020). Recently, we also applied this logic to examine the visual encoding of sex from body shape, revealing a consistent and stimulus-invariant search advantage for female over male body targets (Gandolfo & Downing, 2020). Together, these findings demonstrate the suitability of the search asymmetry approach to study the coding of complex stimuli including objects, bodies and faces.

Here, we report three visual search studies testing the hypothesis that sex is coded asymmetrically, such that the female face is represented as an extension of the male default. Female targets should be easier to find amongst male distractors than *vice versa*, as measured by search rates or by detection accuracy (*d* prime). To ensure that any such effect is generalizable, we tested three kinds of face stimuli: (1) artificially rendered face images; (2) face profile silhouettes, generated from real face source images; and (3) face photographs from four different image databases.

## Methods

### Participants

Participants were students at Bangor University who took part in return for course credit in a research methods module. No individual participant took part in more than one experiment. The procedures were approved by the Research Ethics Committee of Bangor University's School of Psychology, and participants provided written informed consent. The target sample size for each experiment was set at *N* = 32 following our previous work using very similar methods to identify search asymmetries for human body stimuli (Gandolfo & Downing, 2020). Based on the size of the search asymmetry found in our previous work (*d*_z_ = 0.51), with a power analysis we estimated that a sample of 32 participants would be sufficient to detect an effect of a similar size with at least 80% power. In Experiments 1 and 3 we recruited a gender-balanced sample including 16 females in each (age data unavailable for Experiment 1; mean age 20 ± 1.2 years for Experiment 3). Experiment 2 included 23 females (mean age 20 ± 2 years). Participants with overall mean response times or accuracy (averaged across conditions) of >2.5 standard deviations below or above the group mean for that experiment were considered outliers. Their data were excluded and new participants were tested to replace them to reach a sample size of *N* = 32. (For Experiment 2, two additional participants were tested in this phase due to an oversight and their data are included in the present analyses). Exclusion numbers were as follows: 1 in Experiment 1; 3 in Experiment 2; 1 in Experiment 3.

### Stimuli and apparatus

**Fig. 1:**
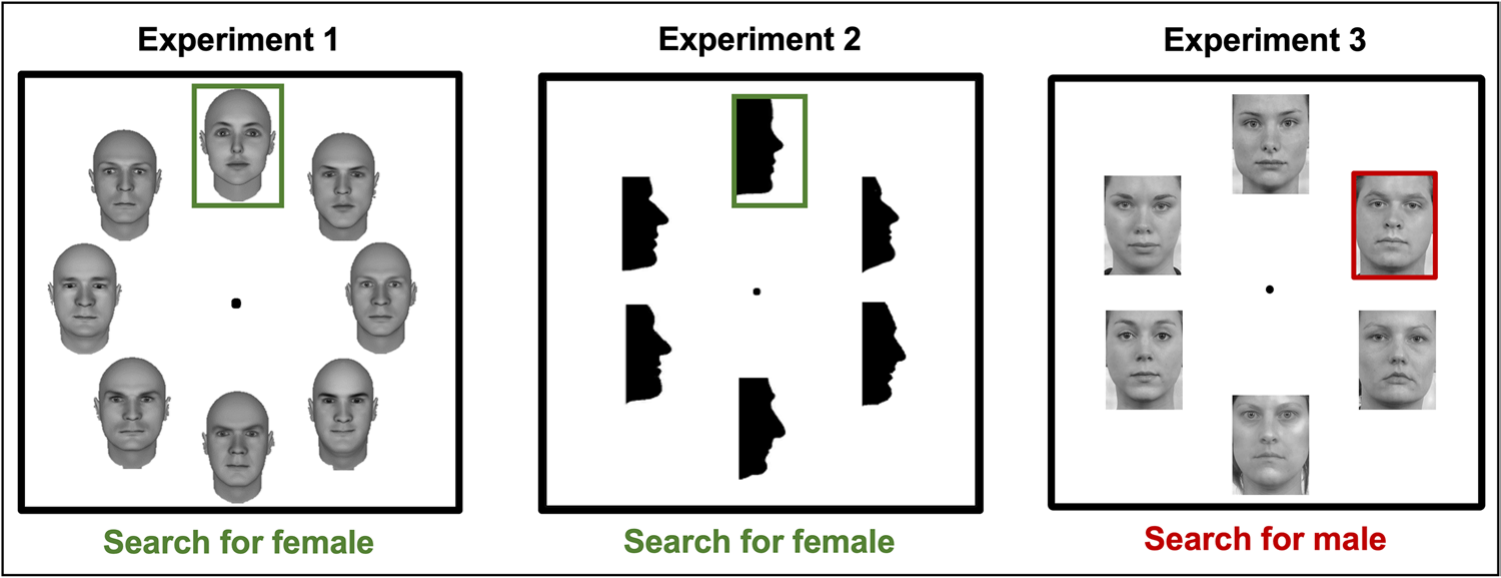
Example stimuli for Experiment 1 (frontal FaceGen faces), Experiment 2 (profile silhouettes) and Experiment 3 (frontal face photographs, here, from the Radboud Faces database; Langner et al., 2010). Search set sizes included 1, 2, 4, or 8 items in Experiment 1, and 1, 2, 4, or 6 items in Experiments 2 and 3. Coloured outlines highlight the target items in each sample display; these were not included in the actual experiments. Task instructions were provided at the start of each block; sample instructions are provided here for illustration only. (Colour figure online)

Experiments were administered using the Psychtoolbox package (Brainard & Vision, 1997; Pelli & Vision, 1997) running in MATLAB (MATLAB Release 2012, The MathWorks, Inc., Natick, MA, USA) on an Apple iMac computer. Viewing distance was approximately 60 cm from the screen but was not fixed. The face images we used are illustrated in Fig. 1. Images from Experiments 1 and 2 are available to download at this link (https://osf.io/ucq2g/). Images from Experiment 3 are available from the maintainers of those face databases (see below).

#### Experiment 1

The faces for Experiment 1 were generated using FaceGen Modeller Version 3.1 (Singular Inversions; Toronto, ON, Canada). Previous studies of face perception have extensively used computer-generated stimuli (e.g., Oosterhof & Todorov, 2008) because they can be manipulated to vary realistically in their high-level social characteristics (sex, age, facial expression, race) while controlling for other low-level visual factors.

We created two sets of stimuli with 40 faces (20 males, 20 females) in each set. Facial expression was set to neutral and the ethnicity was Caucasian for all the faces. The faces were presented without hair. The FaceGen software allows manipulation of sex while keeping the identity and some other dimensions of a face constant (such as expression). Accordingly, the male faces generated for one set were identity matched with the female faces for the other set. Each participant was randomly assigned to perform the experiment with one of the two sets. The faces were scaled to 180 × 180 px each and converted to greyscale.

#### Experiment 2

In Experiment 2, we used face profile silhouettes following Davidenko’s approach (Davidenko, 2007; Davidenko et al., 2012). These stimuli capture the global shape of the face without including confounding internal features such as colour or texture. Previous studies show that face silhouettes are visually processed in many ways like face photographs and provide enough information for accurate age estimation and sex judgments (Davidenko, 2007; Davidenko et al., 2012).

The final sample of silhouettes included 12 images of males and 12 images of females, rendered at 180 × 180 px. Details of the creation, selection, and pixel-level analysis of these stimuli are in the Supplemental Materials.

#### Experiment 3

In Experiment 3, we selected images of neutral-expression faces from four face photograph databases: KDEF (Calvo & Lundqvist, 2008; Goeleven et al., 2008); NimStim (Tottenham et al., 2009); Radboud (Langner et al., 2010); and Stirling (http://pics.psych.stir.ac.uk/ ). From each database, 12 males and 12 females were chosen. The final sample of stimuli included 96 photographs, 48 male and 48 female faces, which were presented at a size of 180 × 140 px.

We matched the selected images for spatial frequency and luminance using the SHINE MATLAB toolbox (Willenbockel et al., 2010; see Supplemental Materials).

### Design and procedure

The design and procedure closely followed Gandolfo and Downing (2020). Participants were instructed in different blocks either to search for a female face amongst male face distractors, or a male face amongst female distractors. The design included four blocks, each comprising 128 trials (Experiments 1 and 2) or 120 trials (Experiment 3); in two blocks the target was male, and in two blocks, female. The four blocks were presented in a counterbalanced order (MFFM or FMMF, equally across participants) with a short break between blocks. In Experiments 1 and 2, within blocks, the trial orders were block randomized such that each chunk of 16 trials consisted of two trials each from the crossing of target (present, absent) by set size. In Experiment 3, each chunk of 32 trials consisted of a counterbalanced combination of source face database, target presence, and set size. In Experiment 1, set sizes varied over 1, 2, 4, or 8 items. In Experiments 2 and 3, set sizes varied over 1, 2, 4, or 6 items.

Each trial started with a central fixation cross of random duration between 800 and 1,200 ms. The search array was presented for 5 seconds or until the participant responded. Each face stimulus could appear randomly in one of the possible equally spaced locations on a virtual circle (radius ~6 cm) around the fixation point (see Supplemental Fig. 1). The target, selected at random from the relevant item set, was present in 50% of the trials. Distractors were randomly chosen without replacement from the relevant image set such that no face distractor could appear more than once in a given trial. Participants were instructed to “press J if a male [female] is present, press F if no male [female] is present” and to respond quickly without sacrificing accuracy.

### Data analysis

Search efficiency was measured by the time required to detect the two target types over varying set sizes, and by sensitivity to detect a target as assessed by d-prime. Search rates were determined by estimating with a linear fit the slope relating search set size to response times (RT) for accurate trials. Smaller values (flatter slopes) reflect more efficient search for the target. Because of the complexities of interpreting target-absent search efficiency relative to target-present performance (e.g., Chun & Wolfe, 1996), we analyzed each separately. Sensitivity was assessed by calculating d-prime. To assess biased decision in search we also analyzed the response bias (β). (See Supplemental Materials for details on how each dependent measure was calculated.)

Because of the close similarity of the procedures for all three experiments, we conducted analyses combining their results, maximizing sensitivity to detect effects of face sex on search efficiency. Specifically, we conducted mixed-design analyses of variance (ANOVAs) with each dependent measure, with sex of target (within participants; male, female) and experiment (between participants) as factors. These were complemented with one-way Bayesian ANOVAs (assessing the effect of Experiment on the difference between male and female search targets) to distinguish the likelihood of true null effects from inconclusive evidence. Separate analyses per experiment can be found in the Supplemental Materials.

Finally, as a planned test of whether participant gender influenced search asymmetries, using the data from Experiments 1 and 3 (in which male and female participants were represented equally), we ran mixed-design ANOVAs on search slopes and *d* prime, with participants’ gender and experiment as between-participants factors, and target sex as a within-participants factor.

## Results

**Fig. 2:**
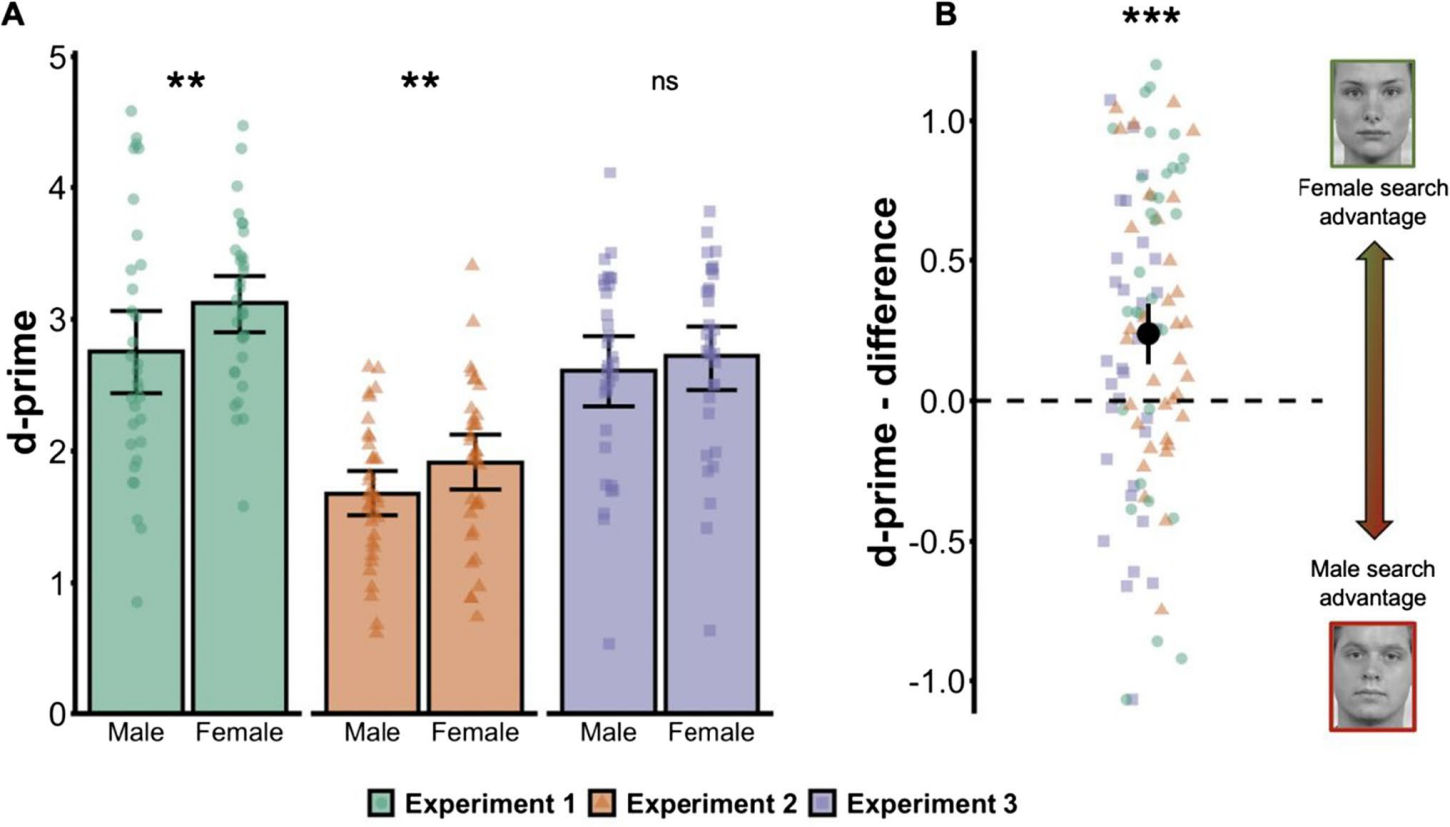
**a** *D* prime as a function of target type in each experiment. Higher values reflect more efficient search for the target (better sensitivity to the difference between target-present and target-absent trials). **b** Point-range plot showing the search asymmetry across experiments as expressed by subtracting male from female targets. Error bars indicate bootstrapped 95% confidence interval of the mean. Individual points represent means for each individual participant in each experiment. *p < .05; **p < .01; ***p < .001. (Colour figure online)

**Fig. 3:**
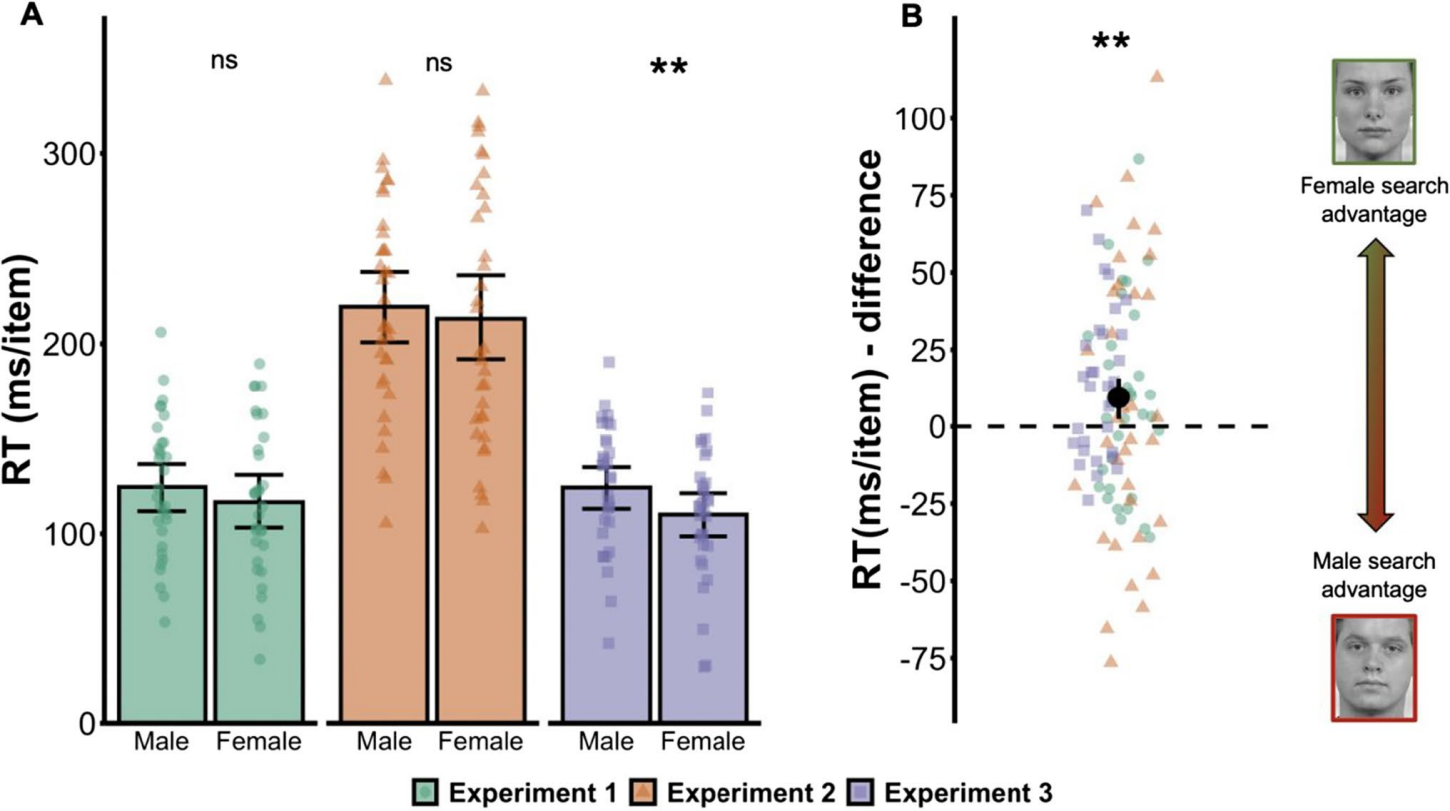
**a** RT search slopes for male and female targets in each experiment. Search slopes were derived from a linear fit to response times on accurate, target-present trials as a function of target type (male or female) and set size. Lower values reflect more efficient visual search (less time required per item to detect the target). **b** Point-range plot showing the search asymmetry across experiments as expressed by subtracting female from male targets. Error bars indicate bootstrapped 95% confidence interval of the mean. Individual points represent means for each individual participant in each experiment. *p < .05; **p < .01; ***p < .001. (Colour figure online)

Mean target-present search slopes and mean *d* primes are reported in Figs. 2 and 3 as a function of target sex. Plots of target absent search slopes and criterion (bias) for each experiment are provided in Supplemental Figs. 2 and 3, and mean accuracy and response times are reported in Supplemental Table 1.

### Target-present RT slopes

The ANOVA on search slopes for accurate target-present trials showed a significant effect of target sex, *F*(1, 95) = 7.02, *p* = .009, Ƞ_p_^2^ = 0.07. Search for female targets (*M* = 147 ms/item, *SD* = 5) was more efficient than search for male targets (157 ms/item, *SD* = 5). We also found a significant main effect of Experiment, *F*(2, 95) = 56.69, *p* < .001, Ƞ_p_^2^ = 0.54: visual search was less efficient for Experiment 2 compared with Experiment 1, *t*(64) = 8.13, *p* < .001, and with Experiment 3, *t*(64) = 8.65, *p* < .001. We did not observe a significant Experiment × Target Sex interaction, *F*(2, 95) = 0.45, *p* = .64, Ƞ_p_^2^ = 0.01, BF_10_ = 0.362.

### Target-absent RT slopes

The ANOVA on search slopes for accurate target-absent trials showed a main effect of search target, *F*(1, 95) = 14.78, *p* < 0.001, Ƞ_p_^2^ = 0.13: reporting the absence of female targets (*M* = 237 ms/item, *SE* = 7.3) was more efficient than for male targets (*M* = 254 ms/item, *SE* = 7.3). There was also a main effect of experiment, *F*(2,95) = 47.59, *p* < 0.001, Ƞ_p_^2^ = 0.50: visual search was less efficient for Experiment 2 compared with Experiment 1, *t*(64) = 6.29, *p* < .001, and with Experiment 3, *t*(64) = 8.91, *p* < .001, and search in Experiment 3 for target-absent trials was more efficient than Experiment 1, *t*(62) = 3.02, *p* = .004. We did not observe a significant interaction between experiment and target sex, *F*(2, 95) = 2.22, *p* = .11, Ƞ_p_^2^ = 0.04, BF_10_ = 1.22.

### Sensitivity (d prime)

The ANOVA on *d* prime showed a main effect of target, *F*(1, 95) = 19.53, *p* < .001, Ƞ_p_^2^ = 0.17. Detection of female targets (*M* = 2.57, *SE* = 2.33) was more accurate than for male targets (2.33, *SE* = 0.07). We also observed a main effect of experiment, *F*(2,95) = 26.71, *p* < .001, Ƞ_p_^2^ = 0.36: detection was less efficient for Experiment 2 compared with Experiment 1, *t*(64) = 7.14, *p* < .001, and with Experiment 3, *t*(64) = 5.73, *p* < .001. Finally, we did not observe a significant interaction between experiment and target sex, *F*(2, 95) = 1.82, *p* = .17, Ƞ_p_^2^ = 0.04, BF_10_ = 0.40.

### Bias

The ANOVA on criterion showed a main effect of target, *F*(1, 95) = 36, *p* < .001, Ƞ_p_^2^ = 0.27, a main effect of experiment, *F*(1, 95) = 9.39, *p* < .001, Ƞ_p_^2^ = 0.17, and a significant Target × Experiment interaction, *F*(1, 95) = 8.53, *p* < .001, Ƞ_p_^2^ = 0.15, BF_10_ = 71.77. Criterion was more conservative in search for female than for male targets (i.e., a male bias) in Experiments 1 and 2 but not in Experiment 3.

### Participant gender

A mixed-design ANOVA on target-present slopes, with participant gender, experiment, and target sex as factors did not show any significant main effects or interactions involving participant gender (all *p*s > .77). The same analysis on d-prime did not show any significant main effects nor an interaction with participant gender (all *p*s > .20). We conducted a two-tailed Bayesian independent-samples *t* test assessing effects of participant gender on a search asymmetry index (subtracting male – female target conditions) for both *d* prime and target-present slopes. The BF_10_ for target-present slopes was 0.27, while for *d* prime it was 0.28, indicating that the null hypothesis was at least three times more likely than the alternative hypothesis, suggesting a true null effect of participant’s gender on the search asymmetry reported here (Jeffreys, 1961; Lee & Wagenmakers, 2013).

### Stimulus heterogeneity

We performed post hoc tests of whether the search benefit for female over male faces is attributable to differences in the homogeneity of the stimuli in the two sets. Search items drawn from a homogenous set will be easier to reject as distractors, compared with a less homogenous set (Duncan & Humphreys, 1989). As an objective test of homogeneity, we used the GIST approach (Oliva & Torralba, 2001) to provide a compact yet physiologically plausible description of the low-level visual features of each face. Similarity between a given pair of faces was construed as the Euclidean distance between the two GIST vectors describing those images, and also the correlation between those vectors. For Experiment 1, both similarity measures indicated reliably greater homogeneity for male than female faces. For Experiment 2, the distance measure indicated greater homogeneity for male faces, but no reliable difference was shown by the correlation measure. For Experiment 3, female images were reliably more homogenous than male images by both measures (see Supplemental Material for details).

## General discussion

We find that observers can detect female faces more efficiently amongst male distractors than vice versa, supporting our hypothesis that coding of sex from the face is polarized rather than balanced. While we did not predict that this advantage would appear variably over experiments in terms of either search rate or detection sensitivity, this variation was not statistically significant, and the search asymmetry was observed over both measures when the data were combined over the three experiments. Further studies could test whether factors such as overall task difficulty, or the use of realistic versus artificial faces, may influence the manifestation of the search asymmetry in measures of speed versus sensitivity. Our findings are unlikely to be due to a single confounding low-level variable, owing to the variety of image formats tested. This does not rule out other possible high-level associations between visual properties and facial sex, which may reflect genuinely valid signals that are used by observers. Our analyses of decision criterion effects also replicated (in two experiments) the male “bias” reported in previous studies.

A polarized coding scheme implies that detectors primarily tuned to the standard (male faces) are more strongly activated by the nonstandard (female faces) than vice versa. In other words, female faces also activate the male detectors, whereas male faces produce less of an effect on female-tuned detectors. (Note we do not equate “detectors” to single neurons; these could instead constitute neural populations). A corollary, identified by Treisman and Gormican (1988) for more elementary visual properties, is that the tuning profiles of these detectors may be different, as illustrated schematically in Fig. 3. The idea is that tuning of detectors for the standard (male) is broader, such that responses are evoked by a wider range of stimulus types: this in part accommodates the male bias at a decisional level. In comparison, detectors that respond to the unique properties of the non-standard (female) are tuned more narrowly. When the nonstandard is a distractor, this generates relatively higher background activity in the detectors for the standard, leading to a difficult target/nontarget discrimination. In contrast, when the nonstandard is a target, it is more detectable by virtue of the additional unique activity over its more narrowly tuned detectors. This description is consistent with computational analyses of how neural populations (in general) most efficiently code stimuli as a function of their frequency. For example, Ganguli and Simoncelli (2014) argue that more frequently-occurring stimuli will be encoded in the activity of relatively more cells with narrower tuning functions compared with less frequent stimuli, thereby increasing the information content of neural activity patterns.

What are the underlying sources of this polarized representation? We posit that experience during early visual development, rather than a decision bias with evolutionary origins, may contribute to such representational asymmetries (see also Gandolfo & Downing, 2020; Quinn et al., 2019 for relevant discussions related to sex; and Furl et al., 2002, for a similar perspective related to race). Visual face representations actively develop within the first year of life (Bhatt et al., 2005; Pascalis et al., 2002). “Sleeper effects” (Maurer et al., 2007) illustrate some of the long-lasting consequences of experience during this early period. For example, infants who are deprived of normal visual input due to congenital cataracts that are corrected within months of birth develop representations of the face that do not, even years later, show the typical hallmarks of configural processing (Le Grand et al., 2001). In Western societies, early caregiving is highly disproportionately provided by adult females (Rennels & Davis, 2008; Sugden et al., 2014; United Kingdom Survey of Childcare and Early Years Providers, 2018), so it follows that the input to early developing face representations is typically mostly from females. This manifests in infancy as an attentional bias for female faces (Ramsey et al., 2005; Righi et al., 2014; see also Rennels et al., 2017). For example, infants aged 3–4 months looked longer at female faces when they were paired together with male faces (Rennels et al., 2017); the preference depended on the participants having females as primary caregivers, reversing in a sample of infants raised primarily by male caregivers.

**Fig. 4:**
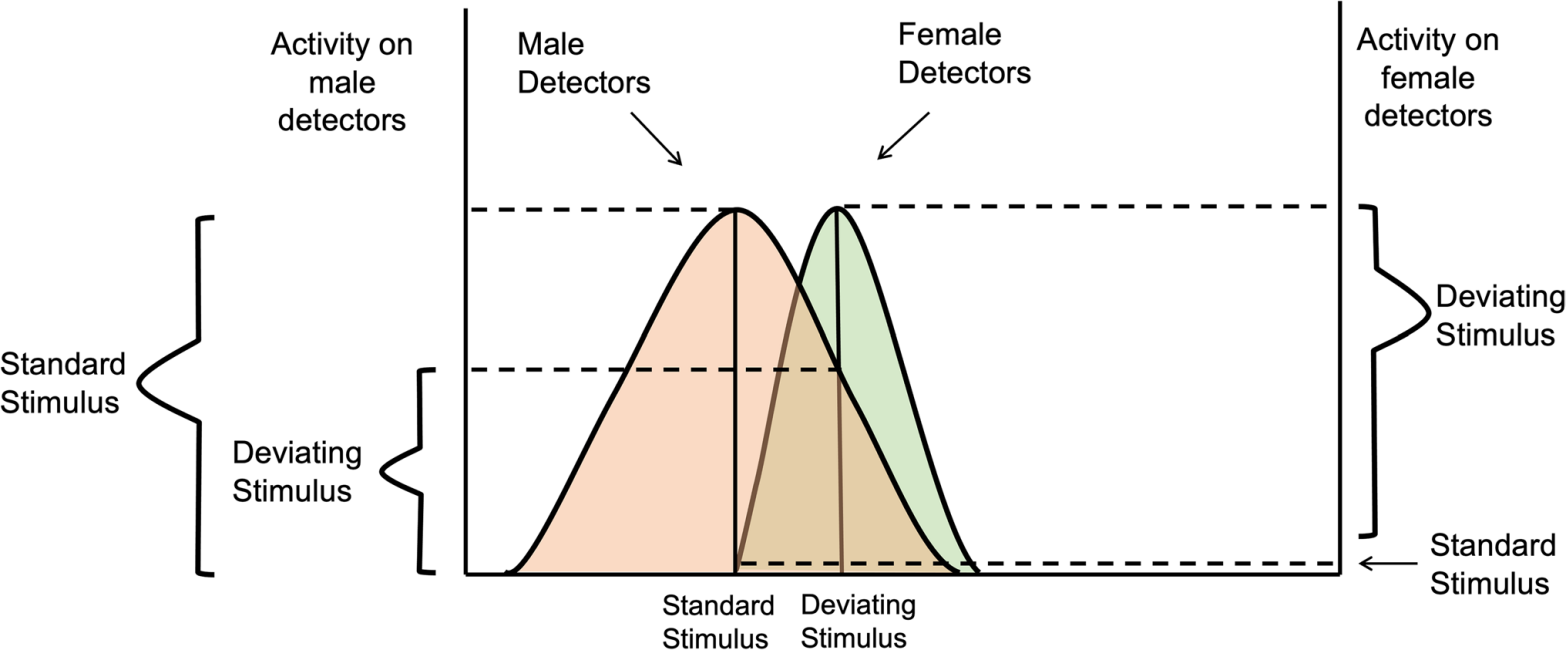
Schematic illustration describing hypothesized perceptual “detectors” that encode sex from facial appearance. Close spacing of peaks reflects high overall similarity of male and female face shapes. Broader tuning for standard (male) than for deviating values (female) accounts in part for an observed “male bias” in that a wider range of stimuli will be judged male than female. A typical female face (peak of orange curve) will elicit some activity on the male detectors and also unique activity on more narrowly tuned female detectors. Conversely, a typical male face (peak of green curve) will activate male detectors and generate little activity on the female detectors. Efficiency of search is governed in part by the ratio of [activity to the target stimulus]:[activity to the distractor stimulus]. Distribution shapes are arbitrary and exaggerated for illustrative purposes. Adapted from Treisman and Gormican (1988). (Colour figure online)

Our proposal is that unbalanced developmental visual experience with female faces results in lasting denser neural encoding of female faces relative to male faces. In the terms of Treisman and Gormican’s (1988) analysis, equates to female “detectors” being more narrowly tuned to their preferred stimuli compared with male “detectors” (Fig. 4), which in turn generates asymmetric search performance for female faces. This proposal does not exclude the influence of other aspects of visual experience on face perception (such as during adolescence; Leder et al., 2003; Picci & Scherf, 2016). Although they are reported in other kinds of face tasks (e.g., Herlitz & Lovén, 2013; Lovén et al., 2011; Scherf et al., 2017), we did not find an influence of participant gender on search asymmetries in the two experiments for which gender was balanced, in line with previous findings for bodies (Gandolfo & Downing, 2020).

We tested for objective differences between our male and female stimulus sets in their homogeneity that could impact search performance. In Experiment 1 the male stimuli were more homogenous than the females, but in Experiment 3 this pattern was reversed. (The results for Experiment 2 were inconsistent; silhouette profiles lack texture and may be poorly suited to a GIST description.) While in future replications it will be useful to closely match stimulus sets on objective or subjective measures of homogeneity, we conclude that objective differences in homogeneity do not fully account for the search asymmetry reported here.

Previously (Gandolfo & Downing, 2020), we used the same visual search strategy to show that for body shape a male bias (Gaetano et al., 2016; Johnson et al., 2012), reflects a deeper asymmetry of perceptual encoding. As here, we argued that these findings reflect a representation of body shape that encodes female body form by reference to a male default. The agreement between faces and bodies is consistent with the developmental experience hypothesis outlined above, although it may also be consistent with others. An open question is whether these analogous findings over faces and bodies reflect a single abstract sex representation, generalizing over face- and body-specific mechanisms (see Ghuman et al., 2010; Palumbo et al., 2015), or instead reflects a common property of distinct domain-specific analyzers. Furthermore, our experience-based account would predict similar polarized coding of sex cues in other dimensions such as body motion patterns, or even voice properties, provided there is evidence that the relevant mechanisms are actively developing during a period in which the “diet” of social experience is typically skewed towards females.

As part of the wider literature on sex and gender perception, our results reveal some of the perceptual processes that rapidly categorize social stimuli, leading to downstream effects on social behaviour (e.g., Hehman et al., 2014). As such, they contribute to broader efforts to understand the interplay between perception, categorization, judgment, stereotypes, and attitudes that takes place constantly in daily life (Freeman & Ambady, 2011; Macrae & Quadflieg, 2010; Todorov, 2017).

## Supplementary information

ESM 1 (DOCX 1.03 MB)

## Data Availability

The datasets generated during and/or analyzed during the current study are available in the OSF repository (https://osf.io/ucq2g/).

## Footnotes

1 In line with current guidance (e.g. https://orwh.od.nih.gov/sex-gender), we use “sex” to refer to a categorical distinction between images of people’s faces and bodies, and “gender” in reference to our participants.
