# Supplementary material for: Asymmetric visual representation of sex from facial appearance
Source: Psychon Bull Rev. 2022 Oct 21;30(2):585–95. doi: 10.3758/s13423-022-02199-6 (PMC10104929; doi:10.3758/s13423-022-02199-6)
Supplement: Supplementary file 1 — (DOCX 1.03 MB) [file 13423_2022_2199_MOESM1_ESM.docx]

**Asymmetric visual representation of sex from facial appearance**

**Marco Gandolfo & Paul E. Downing**

**Supplementary Material**

**Experiment-specific Methods**

**Experiment 2**

**Stimuli**

We first selected and downloaded from the FERET database (Phillips et al., 1998, 2000) 66 profile-view face images (half male, half female). We then generated silhouettes from each face using Adobe Photoshop (Adobe, Inc.). Images were gray-scaled and passed through a threshold filter, resulting in black face profiles on white backgrounds. These two-toned images were then cropped horizontally at the forehead, below the chin, and down the ear line, removing visible hair or clothing, following (Davidenko, 2007).

The 66 silhouettes were each then rated by 29 participants through an online survey performed on Google forms (Google, Inc.). These participants did not take part in the visual search experiments. For each silhouette, participants reported the sex of the silhouette, and indicated how confident they were in their decision on a scale from 1 (not sure) to 5 (sure). Based on the results of this survey, we first selected male and female face silhouettes that were categorised with at least 80% accuracy. Among these accurately judged faces, we then selected those for which participants reported the highest confidence on average.

We also assessed whether the male and female silhouettes in this final sample differed in their size; if so, an asymmetric pattern of search efficiency could potentially be attributed to size differences rather than shape differences related to sex *per se*. An independent samples t-test on the count of black pixels in the images discounted this possibility, t(22) = 0.59, p = 0.56, d = 0.24, CI = [-0.57, 1.04].

**Experiment 3**

**Stimuli**

In Experiment 3, we used face photographs that were processed to obscure the hair and to match images on some low-level features. Each of the face images was cropped so that the hair would not be visible, then converted to grayscale, and then resized to 200x200 px using the R package “Imager” (Barthelmé & Tschumperlé, 2019). The final sample of stimuli included 96 photographs, 48 male and 48 female faces, which were presented at a size of 180 x 140 px.

We then matched the images for spatial frequency and luminance using the SHINE Matlab toolbox (Willenbockel et al., 2010). The equalisation steps were performed in this order: first we matched the luminance histograms of each image with the average luminance distribution of the whole set of images; second, we used the SHINE function sfMatch to equate Fourier amplitudes across stimuli. This function preserves the amplitude distribution across orientations while ensuring that the rotational average amplitudes for a given spatial frequency are equated between images. Preserving the amplitude distribution amongst orientations ensures that the resulting images do not differ too highly from the input images. Importantly, these steps of luminance and spatial frequency matching were performed separately for the faces from each source database (to avoid excessive image degradation), but combined across sex to reduce or remove any systematic lower-level differences between the male and female images chosen. For these reasons, for each trial of Experiment 3, the faces presented were all taken from one of the four source databases, counterbalanced within each block of 24 trials.

**Analyses**

The linear fits for accurate RTs as a function of set size were conducted with the function “lm” in base R (Version: 4.0). Sensitivity was assessed by computing d-prime measures (Macmillan & Creelman, 1990), collapsing over set sizes. Higher d-prime reflects better discrimination of a target present trial from a target absent trial. Extreme hit and false alarm proportions (p = 0 or p = 1) were adjusted according to (Hautus, 1995). The d-prime analysis also determines a measure of response bias (β). The value for β is defined as the ratio of the height of the signal plus noise distribution at the criterion to the height of the noise distribution at the criterion. As β gets larger the observer is said to be more conservative (less likely to report “present”), and as it approaches 0 the observer is considered to be more liberal (more likely to report “present”). Criterion and d-prime were calculated in R (Version 4.0) using the package “Psycho” (Makowski, 2018) where the signal detection theory methods are calculated using the algorithms of (Pallier, 2002).

**Results**

*Analyses per experiment*

We report paired-sample two-tailed t-tests on search slopes in each experiment (note that this is a conservative approach given our directional *a priori* hypothesis of more efficient search for female than male targets). These compared search for male targets amongst female distractors to search for female targets amongst male distractors. Analogous contrasts were applied to the d-prime and criterion measures. For all the t-tests, we report confidence intervals calculated over the difference between male and female target conditions. Results figures per experiment for target present slopes and d-prime are shown in the main text. Results for target absent and criterion measures per experiment are reported in the bar charts in **Supplemental Figure 2, and 3.**

In Experiment 1, the difference between female and male targets on search slopes for accurate target present trials was not significant *t*(31) = -1.46, *p*  = 0.15, d = -0.26, 95% CI = [-0.61, 0.10]. For target absent trials, search for female faces (M = 204 ms/item, SD = 66) was more efficient than search for male faces (M = 235 ms/item, SD = 59, t(31) = -5.06, *p ­*< 0.001, d = -0.89, 95% CI = [-43.40, -18.43]. A paired samples t-test on d-prime showed significantly higher sensitivity when searching for a female target (M = 3.1, SD = 0.64) than a male target (M = 2.8, SD = 0.97), t(31) = 3.32, *p* = 0.002, d = 0.59, 95 % CI = [0.14, 0.59]). A more conservative criterion was observed when searching for a female target (M = 11, SD = 10) than a male target (M = 1.59, SD = 2), t(31) = 5.20, *p* < 0.001, d = 0.92, 95 % CI = [5.72, 13.10]).

In Experiment 2, the difference between female and male targets on search slopes for accurate trials was not significant in target present *t*(33) = -0.79, *p*  = 0.44, d = -0.13, 95% CI = [-22.63,10.02] nor in target absent trials *t*(33) = -0.79, *p*  = 0.43, d = 0.14, 95% CI = [-29.01, 12.75]. A paired samples t-test on d-prime showed significantly higher sensitivity when searching for a female target (M = 1.91, SD = 0.62) than a male target (M = 1.68, SD = 0.53), t(31) = 3.00, *p* = 0.005, d = 0.51, 95 % CI = [0.08, 0.40]). A more conservative criterion was observed when searching for a female target (M = 3.54, SD = 1.30) than a male target (M = 0.60, SD = 0.39), t(31) = 2.47, *p* = 0.02, d = 0.42, 95 % CI = [0.52, 5.36]).

In Experiment 3, there was a significant difference in search slopes for accurate target present trials, *t*(31) = -3.37, *p*  = 0.002, d = -0.60, 95% CI = [-22.79, -5.60]. Search for female targets (M = 110 ms/item, SD = 34) was more efficient than search for male targets (M = 124 ms/item, SD = 33). The same pattern was observed on target absent trials, *t*(31) = -2.18, *p*  = 0.04, d = 0.38, 95% CI = [-26.78, -0.87] where search for female faces (M = 169 ms/item, SD = 60) was more efficient than for male faces (M = 183 ms/item, SD = 54). The paired samples t-test on d-prime did not show a significant difference between male and female search t(31) = -1.25, *p* = 0.22, d = 0.22, 95 % CI = [-0.07, 0.30]), nor did the test on criterion, *t*(31) = 1.91, *p*  = 0.07, d = 0.34, 95% CI = [-0.13, 4.05].

*Analyses of intra-class homogeneity*

We used GIST descriptors to measure, for the faces of each sex in each experiment, all of the unique pairwise similarities between images. Similarity was construed as the Euclidean “distance” between the two vectors describing a pair of images in GIST space. The distributions of these similarities were compared between sexes, to test whether intra-class similarity was significantly greater for images of one sex than the other. This was performed separately for each experiment. To avoid relying on the assumptions of parametric tests, the distributions were compared with bootstrap analyses.

One of the parameters of the GIST analysis defines the number of blocks that form a virtual grid spanning the image. Because GIST was developed mainly with images of scenes in mind, it was not obvious what an appropriate block value would be. Accordingly, GIST analyses of each each experiment were conducted with integer block parameters from 6 (6 x 6 grid over the image) through to 11, inclusive, and the results were concatenated. GIST was computed with the default of 8 orientations per spatial scale, and pre-filtering was performed at the default of 4 cycles per image.

Bootstrap tests were performed on the mean of the similarity distributions with the two.boot function of the simpleboot R package (Peng, 2019). Each test included 1000 bootstrap replicates. For Experiment 1, the mean of GIST distances amongst female images was greater than for males (mean difference 0.012 in arbitrary GIST units) and the two-tailed 95% boundaries of the null distribution [0.004, 0.021] did not include zero, indicating reliably greater homogenity for male than female faces. A similar finding held for Experiment 2: mean difference 0.333, 95% boundaries [0.031, 0.646]. In contrast, for Experiment 3, female images were reliably more homogenous than male images, mean GIST difference -0.196, 95% boundaries [-0.239, -0.151].

Comparable analyses were performed on the data in which homogeneity was assessed by computing the Fisherized correlations between each pair of GIST vectors, rather than Euclidean distances. Means of these values (females – males) indicated more homogenous male stimuli in Experiment 1 (-0.045 [-0.062, -0.026]), no difference in Experiment 2 (-0.004 [-0.111, 0.118]), and more homogenous female stimuli in Experiment 3 (0.051 [0.037, 0.067]).

*
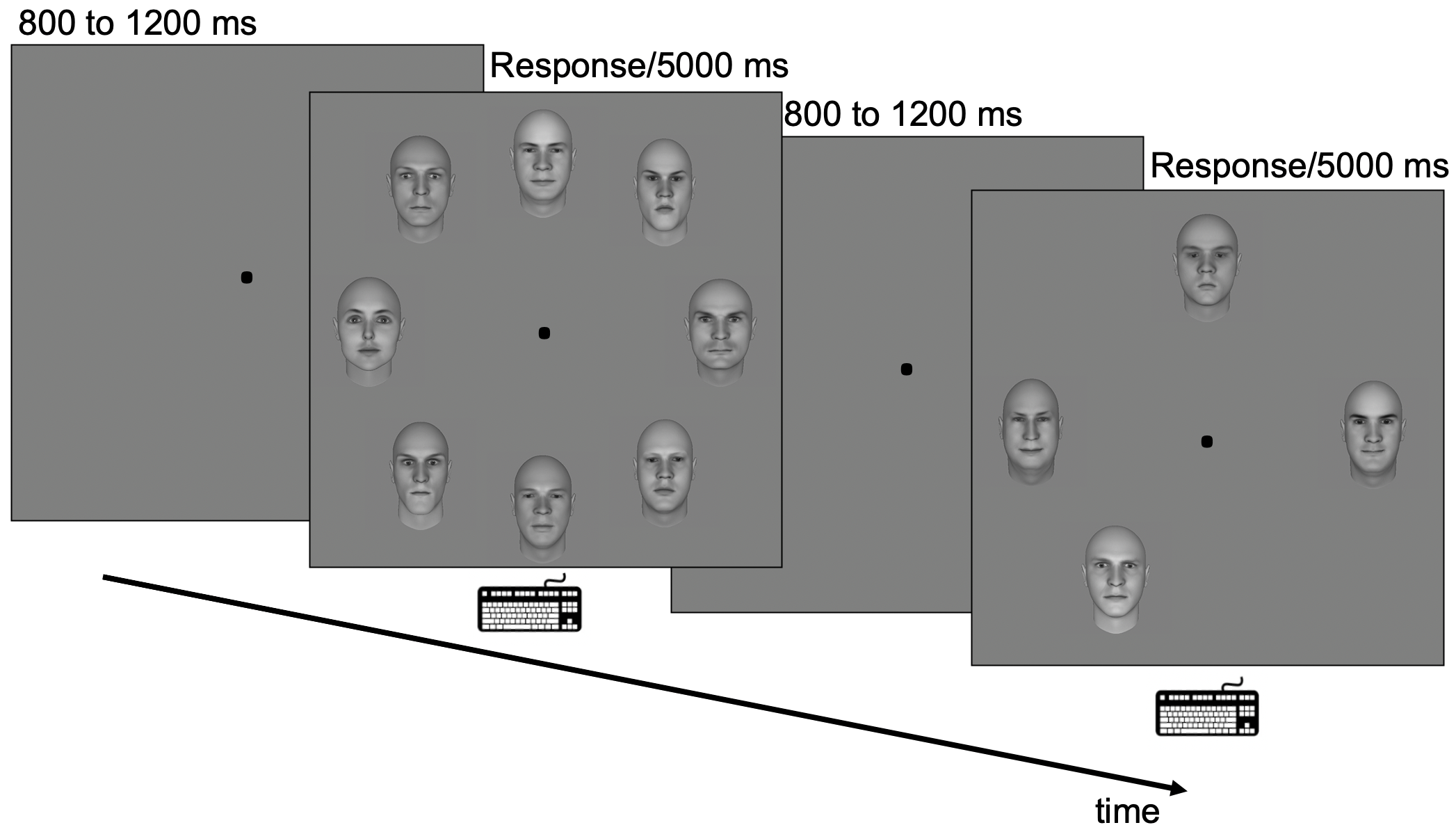
*

**Supplementary Figure 1**. *Illustration of the experimental procedure (with stimuli from Experiment 1). The example shows one target present (set size 8) and one target absent trial (set size 4) of a “Search for female” block.*

**
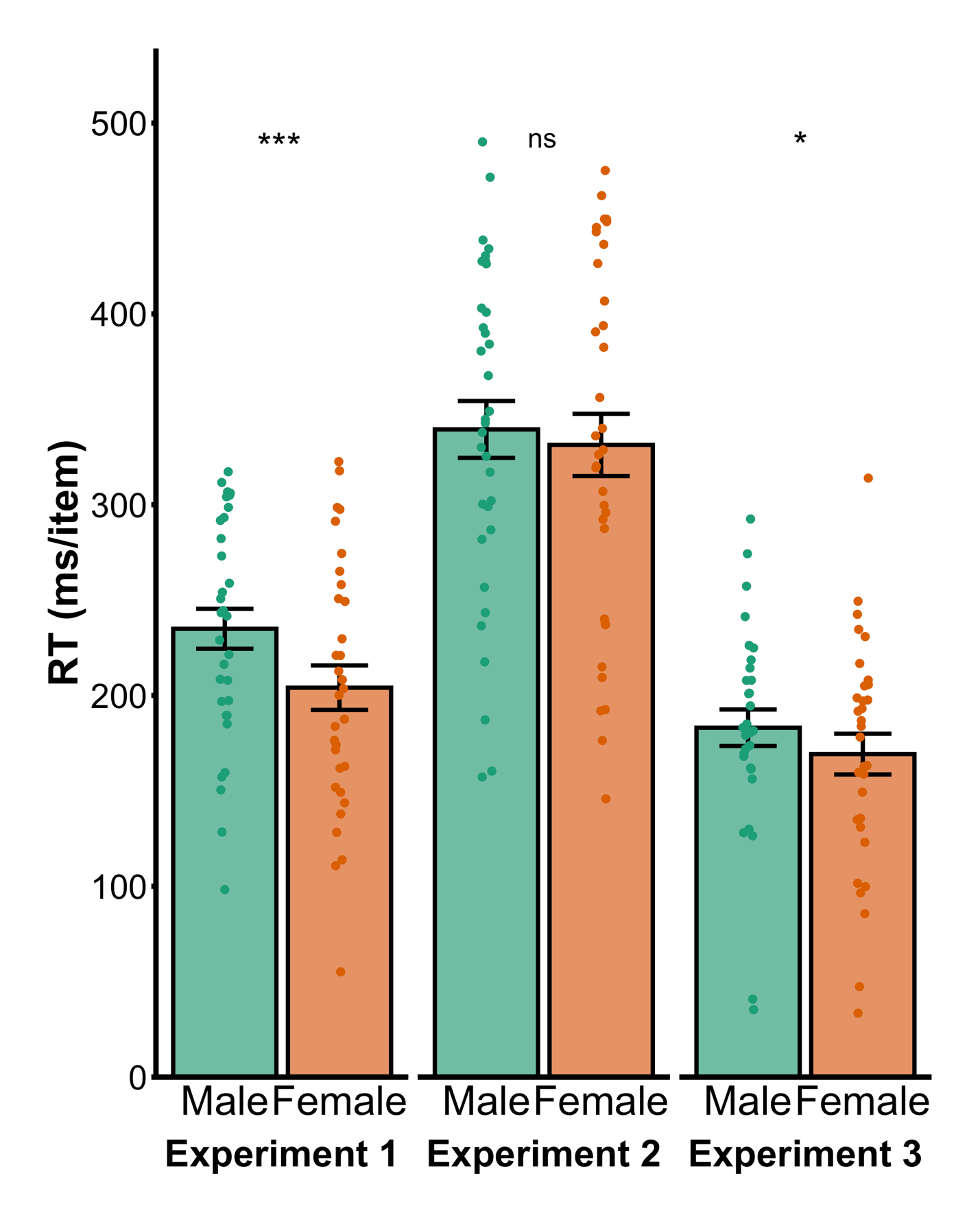
**

**Supplementary Figure 2.** *Target absent slopes effects in search for males amongst females and vice versa. Bars represent grand means over the group, with error bars indicating SE of the mean. Individual points represent means for each individual participant. Asterisks indicate a significant difference between target conditions from a two-tailed paired samples t-test within experiment. * p < .05; ** p < .01; *** p < .001.*

**
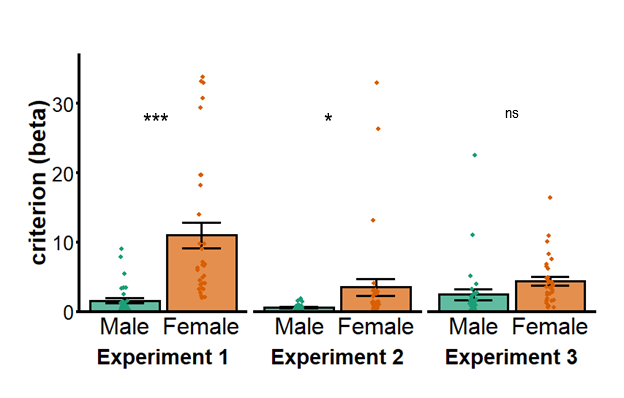
**

***Supplementary Figure 3.*** *Criterion effects in search for males amongst females and vice versa. Conventions as Supplementary Figure 2.*

**Supplementary Table 1.** *Descriptive statistics for accurate RTs as a function of Condition.*

| Experiment Task Target | *M* | *M*  95% CI  [LL, UL] | *SD* |
| --- | --- | --- | --- |
| Experiment 1-Male-Absent | 1494.28 | [1391.47, 1597.08] | 285.13 |
| Experiment 1-Male-Present | 1116.83 | [1042.25, 1191.41] | 206.87 |
| Experiment 1-Female-Absent | 1308.63 | [1215.39, 1401.86] | 258.60 |
| Experiment 1-Female-Present | 1160.40 | [1054.47, 1266.34] | 293.82 |
| Experiment 2-Male-Absent | 1917.22 | [1771.23, 2063.21] | 418.41 |
| Experiment 2-Male-Present | 1510.55 | [1396.18, 1624.92] | 327.79 |
| Experiment 2-Female-Absent | 1757.37 | [1628.30, 1886.44] | 369.91 |
| Experiment 2-Female-Present | 1511.22 | [1388.67, 1633.78] | 351.24 |
| Experiment 3-Male-Absent | 1231.02 | [1155.92, 1306.13] | 208.31 |
| Experiment 3-Male-Present | 1018.04 | [967.05, 1069.02] | 141.41 |
| Experiment 3-Female-Absent | 1145.00 | [1065.42, 1224.58] | 220.73 |
| Experiment 3-Female-Present | 989.50 | [939.09, 1039.91] | 139.81 |

*Descriptive statistics for Accuracy as a function of Condition.*

| Experiment Task Target | *M* | *M*  95% CI  [LL, UL] | *SD* |  |
| --- | --- | --- | --- | --- |
| Experiment 1-Male-Absent | 0.83 | [0.76, 0.89] | 0.18 |  |
| Experiment 1-Male-Present | 0.92 | [0.89, 0.95] | 0.08 |  |
| Experiment 1-Female-Absent | 0.98 | [0.98, 0.99] | 0.02 |  |
| Experiment 1-Female-Present | 0.80 | [0.75, 0.85] | 0.13 |  |
| Experiment 2-Male-Absent | 0.64 | [0.59, 0.70] | 0.16 |  |
| Experiment 2-Male-Present | 0.88 | [0.86, 0.91] | 0.08 |  |
| Experiment 2-Female-Absent | 0.85 | [0.81, 0.89] | 0.11 |  |
| Experiment 2-Female-Present | 0.76 | [0.72, 0.80] | 0.12 |  |
| Experiment 3-Male-Absent | 0.90 | [0.86, 0.93] | 0.09 |  |
| Experiment 3-Male-Present | 0.86 | [0.83, 0.90] | 0.10 |  |
| Experiment 3-Female-Absent | 0.94 | [0.90, 0.97] | 0.10 |  |
| Experiment 3-Female-Present | 0.82 | [0.78, 0.85] | 0.10 | |

**Supplemental References**

Barthelmé, S., & Tschumperlé, D. (2019). imager: An R package for image processing based on CImg. *Journal of Open Source Software*, *4*(38), 1012. https://doi.org/10.21105/joss.01012

Davidenko, N. (2007). Silhouetted face profiles: A new methodology for face perception research. *Journal of Vision*, *7*(4), 6–6.

Hautus, M. J. (1995). Corrections for extreme proportions and their biasing effects on estimated values of d′. *Behavior Research Methods, Instruments, & Computers*, *27*(1), 46–51. https://doi.org/10.3758/BF03203619

Macmillan, N. A., & Creelman, C. D. (1990). Response bias: Characteristics of detection theory, threshold theory, and" nonparametric" indexes. *Psychological Bulletin*, *107*(3), 401.

Makowski, D. (2018). The psycho package: An efficient and publishing-oriented workflow for psychological science. *Journal of Open Source Software*, *3*(22), 470.

Pallier, C. (2002). *Computing discriminability and bias with the R software*. http://www.pallier.org/ressources/aprime/aprime

Peng, R. D. (2019). *simpleboot: Simple Bootstrap Routines* (1.1-7) [Computer software]. https://CRAN.R-project.org/package=simpleboot

Phillips, P. J., Moon, H., Rizvi, S. A., & Rauss, P. J. (2000). The FERET evaluation methodology for face-recognition algorithms. *IEEE Transactions on Pattern Analysis and Machine Intelligence*, *22*(10), 1090–1104.

Phillips, P. J., Wechsler, H., Huang, J., & Rauss, P. J. (1998). The FERET database and evaluation procedure for face-recognition algorithms. *Image and Vision Computing*, *16*(5), 295–306.

Willenbockel, V., Sadr, J., Fiset, D., Horne, G. O., Gosselin, F., & Tanaka, J. W. (2010). Controlling low-level image properties: The SHINE toolbox. *Behavior Research Methods*, *42*(3), 671–684. https://doi.org/10.3758/BRM.42.3.671
